# Analyzing RBC Transfusion Practices Using Quality Indicators: A Retrospective Transfusion Audit

**DOI:** 10.7759/cureus.69550

**Published:** 2024-09-16

**Authors:** Arunpreet Kaur, Dnyaneshwar Patale, Trupti Lokhande

**Affiliations:** 1 Transfusion Medicine, All India Institute of Medical Sciences, Raebareli, Raebareli, IND

**Keywords:** crossmatch to transfusion ratio, quality indicators, red blood cell transfusion practices, retrospective audit, transfusion audit, transfusion index, transfusion probability, utilization rate

## Abstract

Objectives

Overordering of blood products, particularly packed red blood cells (PRBC), leads to inefficiencies and financial burdens within healthcare systems. The objective of this audit was to assess PRBC utilization practices against established quality indicators to enhance efficiency and reduce wastage in a newly established tertiary care hospital in Northern India.

Materials and methods

A retrospective audit was conducted in the department of transfusion medicine. Data were collected from departmental records and analyzed using various quality indicators, such as crossmatch to transfusion ratio (CTR), transfusion probability (%T), transfusion index (TI), and utilization rate (UR). Microsoft Excel was utilized for statistical calculations including range, percentage, ratio, and quality indicators.

Results

A total of 1,488 PRBC requisitions were received, 997 (67%) originated from various surgical specialties. The overall CTR was 1.88, with medical and surgical specialties having CTRs of 1.3 and 2.6, respectively. Overall %T was 53.8%, which in medical departments was 85% and in surgical departments was 38.5%. TI was 0.68 overall, which varied among medical (1.1) and surgical (0.48) specialties. The overall UR was 53.14%. Peri-operative blood loss (48%) and anemia (46%) were the primary indications for transfusions. Cardiothoracic and vascular surgery (CTVS), General surgery, and orthopaedics exhibited appropriate blood utilization practices, whereas ear nose and throat (ENT), pediatric surgery, urology, neurosurgery, and obstetrics and gynecology (OBGY) showed tendencies toward overordering and underutilization.

Conclusion

This audit highlights significant issues related to blood utilization practices, particularly overordering and underutilization in certain surgical specialties within the studied institution. While these findings underscore the potential benefits of implementing audit-driven policies to enhance efficiency and reduce wastage, the results are specific to this institution and may not be universally applicable. Further studies across multiple institutions are recommended to validate these findings and develop broader guidelines for optimizing blood utilization in healthcare systems.

## Introduction

Blood is a valuable but scarce resource in healthcare settings. To date, there is no substitute for blood that possesses all the properties of human blood. In the current scenario, both developed and developing countries, such as India, face challenges in maintaining an adequate and timely supply of safe blood to meet clinical demand. India’s demand for blood is around 14.1 million blood units (1% of the population as per WHO) and is constantly increasing [1]. However, according to a recent study by Mammen et al., there is a gap of around one million units between demand and supply (supply is 13.5 million units) [2].

The blood requisition in elective and emergency procedures from different departments in any multidisciplinary hospital is often associated with excessive demands for cross-matching of blood and blood products. Many previous studies have also shown that there is often over-ordering of blood by surgeons and clinicians [3]. Surgeons prefer to reserve blood for all surgical patients, irrespective of their hemoglobin levels, due to the fear of inadvertent intra-operative bleeding [4]. But over-ordering leads to the non-availability of cross-matched units for other patients who might need blood, as they are kept reserved for patients whose transfusion requirements are uncertain. Reservation of blood leads to loss of shelf life and wastage. It also increases the financial burden on transfusion service providers in terms of storage costs, reagent wastage, time, and human resources. Over-ordering of blood also increases costs for patients during hospital stays [5].

Several indices can be used in transfusion services to check the efficacy of blood ordering and utilization systems. Henry et al. were the first to recommend, in the 1970s, the use of the crossmatch-to-transfusion (CTR) ratio. Ideally, this ratio should be 1.0, but a ratio of 2.5 and below was advocated to be demonstrative of efficient blood usage [6]. Apart from the CTR, other indices are the transfusion probability (%T) and transfusion index (TI), with target values of ≥30% and ≥0.5, respectively [7].

These quality indicators can be used to review a healthcare facility’s blood ordering and transfusion practices, decreasing wastage of blood, reagents, time, human resources, and most importantly, the financial burden on patients. Hence, to analyze blood ordering and transfusion practices at our center, we planned to review quality indicators for better blood management.

Aims and objectives

This study was designed with the aim to review, analyze, and improve the trends of red cell ordering, issuing, utilization, and wastage of packed red blood cells (PRBCs) by various medical and surgical specialties of our hospital by comparing them with the target values of various quality indicators.

## Materials and methods

This was a retrospective single-center study conducted in the Department of Transfusion Medicine at a 600-bed tertiary care hospital in Northern India. Approval from the institutional ethics committee (F3/BIOETHICS/AIIMS-RBL/APPR/IMP/2024-6/1 dated 21-02-2024) was obtained for the study. Data were collected from September 2022 to June 2023. Only PRBC requisitions from various specialties of our hospital were included in the study. Data were collected from patient blood component requisition forms, compatibility, and issue registers. Data were retrieved in the form of age, gender, blood group, department, and indication for transfusion, pre-transfusion hemoglobin, number of PRBC units requested, number of PRBC units crossmatched, number of PRBC units issued, number of PRBC units transfused, and number of PRBC units returned to the blood center within 30 minutes and after 30 minutes of issue as per the institutional return policy. The various quality indicators for blood utilization studied were calculated as follows:

Crossmatch-to-transfusion ratio (CTR) = number of units crossmatched/number of units transfused

It is used to monitor the appropriateness of blood component ordering. A lower ratio typically suggests better blood utilization efficiency, indicating that fewer crossmatches are performed per unit of blood transfused. Conversely, a higher ratio may indicate over-ordering of blood products, wastage, or inappropriate transfusion practices.

Transfusion probability (%T) = number of patients transfused x 100/number of patients crossmatched

It is the probability of blood being transfused in each surgical procedure, and its value ≥ 30% suggests significant blood utilization. It depends on the number of patients crossmatched and the number of patients transfused and is independent of the number of blood units crossmatched or transfused, i.e., CTR.

Transfusion index (TI) = number of units transfused/number of patients transfused

It helps to assess the proportion of crossmatched PRBC units that are transfused to patients. A higher TI indicates that a significant portion of the blood units crossmatched are indeed utilized for transfusions, indicating more efficient blood utilization practices.

Utilization rate (UR) = number of units transfused x 100/number of units crossmatched

It is used to assess the efficiency of blood product utilization. A higher utilization rate indicates more efficient utilization of blood products, as a larger proportion of the crossmatched units are transfused to patients. Conversely, a lower utilization rate suggests inefficiencies in blood product utilization, such as over-ordering, wastage, or inappropriate transfusion practices.

Statistical analysis

For data analysis, Microsoft Excel was employed for calculating range, percentage, ratio, and quality indicators.

## Results

A total of 1908 PRBC units were demanded for 1488 patients in the requisition forms received during the study period. Requisitions were divided into medical and surgical specialties, as shown in Tables 1-2, respectively. 

**Table 1:** Requisitions from medical specialties. In medical specialties, the highest number of requests was raised by general medicine, and the fewest by cardiology.

S. No.	Medical specialties	No. of patients requiring packed red blood cells (PRBC)	Percent
1	General Medicine	352	71.7
2	Pediatrics	59	12.0
3	Medical Intensive Care Unit (MICU)	56	11.4
4	Pediatric Intensive Care Unit (PICU)	13	2.6
5	Cardiology	11	2.2
	Total	491	

**Table 2:** Requisitions from surgical specialties. In surgical specialties, the highest number of requests was raised by the Obstetrics and Gynecology Department, and the fewest by the ENT Department.

S. No.	Surgical specialties	No. of patients requiring packed red blood cells (PRBC)	Percent
1	Obstetrics and gynecology (OBGY)	395	39.6
2	Neurosurgery	199	20.0
3	General surgery	129	12.9
4	Orthopaedics	102	10.2
5	Urology	72	7.2
6	Pediatric surgery	64	6.4
7	Cardiothoracic and vascular surgery (CTVS)	26	2.6
8	Ear nose and throat (ENT)	10	1.0
	Total	997	

**Figure 1:**
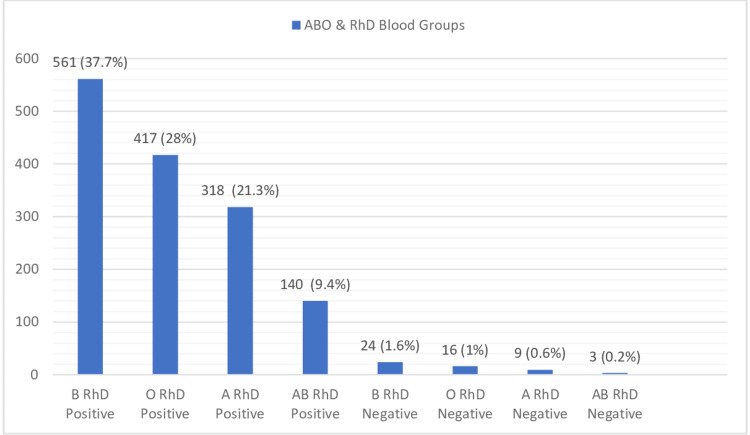
ABO and RhD blood groupwise distribution. Out of 1488 individuals, 1436 (96.5%) were RhD positive and 52 (3.5%) were RhD negative. Among the ABO blood groups, the most common was B, followed by O, A, and AB.

The majority of patients in all specialties required one unit of PRBC, followed by two, three, and four units. A total of 55.5% of patients were females, and 44.5% were males. The age of the patients ranged from three days to 95 years, with hemoglobin levels ranging from 1.5 gm/dl to 17.6 gm/dl. Pre-transfusion hemoglobin was not mentioned in 41 (2.7%) of the requisition forms. Also, the indication for packed red cell transfusion was not mentioned in 66 (4.4%) of the cases. The ABO and RhD blood group distribution of the study population is shown in Figure 1.

Quality indicators were calculated for medical and surgical specialties and then collectively (Tables 3-5).

**Table 3:** Quality indicators in medical specialties. In all medical departments, blood utilization indices were well within the target range, with the Pediatric Intensive Care Unit (PICU) showing the best utilization. The analysis indicated that red cell ordering, issuing, and utilization practices were proper across all medical specialties in our hospital, with minimal wastage. CTR: Crossmatch-to-transfusion ratio [6]; %T: Transfusion probability [7]; TI: Transfusion index [7]; UR: Utilization rate. An asterisk (*) indicates that the target value for the utilization rate has not been defined in the literature. While a few studies have calculated the utilization rate, the target value has not been discussed [8].

S. No.	Medical specialties	Number of PRBC units		Number of patients		CTR	%T	TI	UR
		Cross-matched	Transfused	Cross-matched	Transfused				
1	Cardiology	15	13	11	10	1.2	90.9	1.2	86.7
2	Pediatric Intensive Care Unit (PICU)	13	12	13	11	1.1	84.6	0.9	92.3
3	Medical Intensive Care Unit (MICU)	81	61	56	40	1.3	71.4	1.1	75.3
4	Pediatrics	65	53	59	49	1.2	83	0.9	81.5
5	General Medicine	501	393	352	307	1.3	87.2	1.1	78.4
	Total	675	532	491	417	1.3	84.9	1.1	78.8
Target value of indices						≤2.5 [6]	≥30% [7]	≥0.5 [7]	Not Defined*

**Table 4:** Quality indicators in surgical specialties. In surgical specialties and super-specialties, blood utilization indices varied so widely with general surgery department showing best and ENT department showing worst red cell ordering, issuing and utilization practices. CTR: Crossmatch-to-transfusion ratio [6]; %T: Transfusion probability [7]; TI: Transfusion index [7]; UR: Utilization rate. An asterisk (*) indicates that the target value for the utilization rate has not been defined in the literature. Although a few studies have calculated the utilization rate, the target value has not been discussed [8].

S. No.	Surgical Specialties	Number of PRBC units		Number of patients		CTR	(%T)	TI	UR
		Cross-matched	Transfused	Cross-matched	Transfused				
1	Ear Nose and Throat (ENT)	15	2	10	2	7.5	20	0.20	13.3
2	Cardiothoracic and vascular surgery (CTVS)	48	28	26	18	1.7	69.2	1.08	58.3
3	Pediatric Surgery	69	27	64	28	2.6	43.7	0.42	39.1
4	Urology	76	11	72	10	6.9	13.8	0.15	14.4
5	Orthopaedics	124	69	102	56	1.8	54.9	0.68	55.6
6	Gen Surgery	185	137	129	99	1.4	76.7	1.06	74
7	Neuro Surgery	269	79	199	60	3.4	30.1	0.40	29.3
8	Obstetrics and Gynecology (OBGY)	447	129	395	111	3.5	28.1	0.33	28.8
	Total	1233	482	997	384	2.6	38.5	0.48	39
Target value of indices						≤2.5 [6]	≥30% [7]	≥0.5 [7]	Not Defined*

**Table 5:** Quality indicators in medical and surgical specialties combined. Of the total PRBC requests, 67% were raised by surgical departments, while 33% were raised by medical departments. The cumulative quality indices from both types of specialties indicate efficient blood utilization. CTR: Crossmatch-to-transfusion ratio [6]; %T: Transfusion probability [7]; TI: Transfusion index [7]; UR: Utilization rate. Two asterisks (**) indicate that the target value for the utilization rate has not been defined in the literature. Although a few studies have calculated the utilization rate, the target value has not been discussed [8].

S.N.	Speciality	Number of PRBC units		Number of patients		CTR	%T	TI	UR
		Cross-matched	Transfused	Cross-matched	Transfused				
1	Medical	675	532	491 (33%)	417	1.3	84.93	1.1	79
2	Surgical	1233	482	997 (67%)	384	2.6	38.52	0.48	39
	Total	1908	1014	1488	801	1.9	53.8	0.68	54
Target value of indices						≤2.5 [6]	≥30% [7]	≥0.5 [7]	Not defined*

Quality indicators were further evaluated according to different indications for PRBC transfusion (Table 6).

**Table 6:** Indication-wise comparison between quality indicators. According to the indications mentioned on the PRBC requisition forms, the most common indication was peri-operative blood loss (48%), which included pre, intra, and post-operative bleeding, followed by anemia (46%). In only 2% of cases, PRBCs were demanded for patients with active bleeding. It was found that the indication was not mentioned in 4% of cases. CTR: Crossmatch-to-transfusion ratio [6]; %T: Transfusion probability [7]; TI: Transfusion index [7]; UR: Utilization rate. An asterisk (*) indicates that the target value for the utilization rate has not been defined in the literature. Although a few studies have calculated the utilization rate, the target value has not been discussed [8].

S. No.	Indication	Number of PRBC units		Number of patients		CTR	%T	TI	UR
		Cross-matched	Transfused	Cross-matched	Transfused				
1	Peri-operative blood loss	856	212	710	171	4.04	24.08	0.30	24.77
2	Anemia	928	737	683	581	1.26	85.07	1.08	79.42
3	Not mentioned	87	44	66	30	1.98	45.45	0.67	50.57
4	Active bleed	37	21	29	19	1.76	65.52	0.72	56.76
	Total	1908	1014	1488	801	1.88	53.83	0.68	53.14
Target value of indices						≤2.5 [6]	≥30% [7]	≥0.5 [7]	Not Defined*

The CT ratio for peri-operative blood loss was 4.04, with a transfusion probability of only 24%. The transfusion index was also below the target value. In contrast, the CT ratio, transfusion probability, and transfusion index for other indications were indicative of efficient blood utilization, with utilization rates of 79% and 56% for anemia and active bleed, respectively. In 4% of cases, where the indication was not mentioned, the quality indices showed adequate utilization of blood.

Also, the mean hemoglobin in patients with anemia was 6 gm/dl, in peri-operative patients was 11.8 gm/dl, in patients with active bleed was 9.5 gm/dl, and in cases where the transfusion indication was not mentioned was 9 gm/dl.

A huge deviation from the target value of different QIs was seen in peri-operative bleeding, so it was further elaborated as per different surgical specialties (Table 7).

**Table 7:** Comparison of quality indicators in different surgical specialties in case of peri-operative blood loss. Only the CTVS, General Surgery, and Orthopedics departments demonstrated appropriate blood utilization practices. The ENT, Pediatric Surgery, Urology, Neurosurgery, and OBGYN departments exhibited over-ordering and lower utilization practices. CTR: Crossmatch-to-transfusion ratio [6]; %T: Transfusion probability [7]; TI: Transfusion index [7]; UR: Utilization rate. An asterisk (*) indicates that the target value for the utilization rate has not been defined in the literature. While a few studies have calculated the utilization rate, the target value has not been discussed [8].

S. No.	Surgical Speciality	Number of PRBC units		Number of patients		CTR	%T	TI	UR
		Cross-matched	Transfused	Cross-matched	Transfused				
1	Ear Nose and Throat (ENT)	9	0	6	0	Undefined	0	0	0
2	Cardiothoracic and vascular surgery (CTVS)	32	18	15	11	1.78	73.3	1.2	56.2
3	Gen Surgery	46	21	35	16	2.19	45.7	0.6	45.6
4	Pediatric Surgery	49	10	43	11	4.90	25.5	0.2	20.4
5	Orthopaedics	67	30	59	25	2.23	42.3	0.5	44.7
6	Urology	66	5	64	5	13.20	7.8	0.08	7.5
7	Neuro Surgery	248	60	186	51	4.13	27.4	0.3	24.1
8	Obstetrics and Gynecology (OBGY)	339	68	302	56	4.99	18.5	0.2	20
	Total	856	212	710	175	4.04	24.6	0.3	24.7
Target value of indices						≤2.5 [6]	≥30 [7]	≥0.5 [7]	Not Defined*

## Discussion

Blood transfusion is one of the most frequently performed procedures in the hospital as a life-saving measure. Contrary to this, blood transfusion has also been identified as one of the most commonly overused interventions. What makes these two already risky combinations of high frequency and overuse even more dangerous is an overall complex process involving a multidisciplinary group of healthcare professionals with varying levels of understanding of blood transfusion practice. Frequent checks or audits in blood centers are a very good tool to assess blood utilization practices in multidisciplinary healthcare settings. It is a necessary tool for better patient care and PBM for preventing unnecessary wastage of blood, time, resources, and human resources.

We conducted a retrospective audit aimed to review, analyze, and improve the trends of red cell ordering, issuing, utilization, and wastage of PRBCs by various medical and surgical specialties of our hospital. It was observed that about two-thirds of requests for PRBC were raised by surgical specialties, and the rest of the requests (34%) were raised by medical specialties. Overall, a total of 1908 PRBC units were requested for 1488 patients. For the majority of patients (1123), a single PRBC unit was demanded by various specialties; however, for a few patients, up to four units were requested. As per the stock available in our blood center, all 1908 PRBC units were crossmatched and kept reserved. A total of 1036 units were requested for release; however, only 1014 units were transfused, and 22 units were sent back to the blood center. Out of the 22 units received back, 18 were received within 30 minutes and thus taken back into the inventory. Four units were received back after 30 minutes and were not taken into the inventory but were discarded by the Transfusion Medicine Department according to departmental policy and Bio-Medical Waste (BMW) Management Rules, 2016. This is also in accordance with the guidelines published by the British Committee for Standards in Haematology in 2009 [9].

We also evaluated the indices frequently used in the transfusion services to check the efficacy of blood ordering and utilization practices. The individual indices are discussed as follows:

CTR

The overall CTR was 1.88; however, when segregated by specialty, the CTR of medical specialties was 1.3 and that of surgical specialties was 2.6. The target CT ratio was ≤ 2.5. Higher CTRs have also been reported in other studies among surgical departments and indicated as the best quality indicator for estimating over-ordering or under-utilization of PRBC units [10-12].

CTR was also evaluated among different surgical specialties. It was lowest in general surgery (1.4) followed by CTVS (1.7) and orthopedics (1.8), which was below the target value of 2.5, suggesting adequate blood ordering and utilization practices by these departments. In the rest of the surgical departments, it ranged from 2.6 (in pediatric surgery) to 7.5 (in ENT). Very few surgical procedures in these specialties required blood, as indicated by the high CTR in these fields. However, there is a practice of ordering blood prior to every surgery without any audit-based estimation. Similar findings were also reported in previous studies from India [8,13].

A high CTR in surgical departments suggests that most surgeons order PRBCs when they fear intraoperative bleeding, ensuring that they will have timely access to PRBCs in case of an unforeseen demand. To overcome this apprehension of not getting timely blood in an emergency, we may adopt a type-and-screen (TS) with immediate spin (IS) crossmatch policy instead of the conventional anti-human globulin (AHG) crossmatch and reserve policy to supply blood in all elective surgeries and routine transfusions [6]. TS with IS crossmatch policy has been adopted by various national and international blood centers. Studies done by Alavi-Moghaddam [14], Chow [15], Alexander and Henry [16], and Kuriyan [17] have concluded that the implementation of TS policy has been proven to be efficient and beneficial to the transfusion practice in their respective hospitals.

A study conducted by Aggarwal G et al. in India highlighted that the TS policy resulted in a notable reduction in the CTR, from 1.94 to 1.04, as well as a decrease in PRBC issue turnaround time (TAT), with an average of 65.62 minutes compared to 79.71 minutes in the conventional policy. Moreover, the TS policy led to the elimination of RBC outdating during the study. Man-hours were also saved due to fewer crossmatches and less need for reservation management, which allowed technicians to focus on other tasks. Additionally, monetary savings were achieved, with a 33% reduction in costs compared to the conventional policy, as fewer crossmatches were performed and fewer reagents were used. These findings demonstrate that the TS policy with IS crossmatch is not only safe but also more efficient and cost-effective, benefiting blood banks, hospitals, and patients alike [18].

Transfusion probability (%T)

The combined probability in our study was 53.8%, which meant that the probability of transfusion in our patients was high. This finding is similar to the study by Subramanian A et al. (2012) and Raghuwanshi B B et al. (2017), in which %T was >50% [19,20]. In medical departments, it was relatively high (85%) compared to surgical departments (38.5%). The most common indication for transfusion in medical specialties was anemia. In surgical patients, the most common indication was peri-operative bleeding. The relatively lower %T in surgical departments (38.5%) indicates that only a minority of patients undergoing surgical procedures experienced significant blood loss requiring transfusion, particularly with OBGYN patients, which highlights their concerns like postpartum hemorrhage.

Transfusion index (TI)

The overall TI was 0.68, with values of 1.1 and 0.48 observed in patients of medical and surgical specialties, respectively. In medical departments, TI varied from 0.9 (in PICU) to 1.2 (in cardiology). In surgical departments, it ranged from 0.15 (in urology) to 1.08 (in CTVS). TI was lowest in urology (0.15) followed by ENT (0.2), OBGYN (0.3), neurosurgery (0.4), and pediatric surgery (0.42), indicating fewer transfusions per patient in these departments. Ebose EM et al. reported a similar range of TI (0.1-0.4) in their study in surgical departments [21]. However, in other surgical departments (pediatric surgery, orthopedics, general surgery, and CTVS), it was well above the target value of 0.5. According to different indications, TI was lowest (0.3) in peri-operative blood loss and highest in anemia (1.08). These indications were also similar in a previous study [22].

Utilization rate (UR)

The overall UR in all specialties combined was 53.14%. The target value for the utilization rate has not been defined in the literature; however, in the recent study conducted by Tripathi PP (2023), the overall utilization rate in his study was 59.8% [8]. On broader segregation, UR was found to be higher in medical specialties (79%) compared to surgical (39%), indicating that a greater proportion of blood units reserved for medical departments were utilized for transfusions, whereas a smaller proportion of reserved blood units in surgical departments were utilized. This suggests a more efficient utilization of blood units in medical departments compared to surgical specialties. The lower utilization rate and higher percentage of unutilized blood units in surgical departments indicate a need for optimizing blood ordering and utilization practices in these departments to minimize wastage and ensure efficient use of blood resources.

Quality indicators were also analyzed according to the indication, where indications for PRBC transfusions were categorized into peri-operative blood loss, anemia, active bleed, and a fourth category consisting of unknown indications not specified in requisition forms. Blood utilization was maximum in anemic patients (79%) followed by active bleed (56%) and least in peri-op blood loss (25%). This pattern highlights the critical importance of addressing preoperative anemia correction and intraoperative bleeding management in our patient population. Preoperative anemia is a common yet under-addressed issue that can lead to increased transfusion requirements and poorer surgical outcomes. Implementing standardized protocols for early detection and management of anemia, including the use of iron therapy, erythropoiesis-stimulating agents, and other hematinic interventions, can significantly reduce the need for transfusions in the perioperative period. Additionally, incorporating patient blood management (PBM) strategies that focus on minimizing intraoperative blood loss, such as the use of antifibrinolytics, meticulous surgical techniques, advanced hemostatic agents, and autologous blood salvage, can further reduce reliance on blood products. The involvement of a transfusion specialist is crucial in coordinating these efforts, ensuring appropriate blood utilization, and integrating PBM principles into clinical practice. This comprehensive approach not only enhances patient outcomes but also contributes to more efficient use of blood resources, reducing both wastage and healthcare costs [23].

There were few departments where the utilization rate was as low as 10%. The ENT department did not utilize a single unit for surgical procedures. The higher rates of over-ordering and under-utilization of crossmatched units in the peri-operative period unmistakably indicate a pattern rather than coincidence, but this is due to surgeons’ habit of requesting the reservation of PRBC units prior to any surgery without any rationale of a maximum surgical blood ordering schedule (MSBOS) [24].

An MSBOS is a schedule of commonly performed elective surgical procedures listing the maximum number of units of blood to be crossmatched preoperatively. An MSBOS reduces the preoperative crossmatching of blood in surgical cases in which there is less likelihood of blood transfusion. The reduction in crossmatched units of blood saves hospital time and money [25].

Vibhute M et al. showed an improved utilization rate from 23.14% to 74.74% after the implementation of MSBOS [26]. By using this audit data, we can suggest the hospital transfusion committee (HTC) of our institute prepare and implement MSBOS for elective surgical procedures [27]. To decrease wastage of blood due to the reservation of blood for a prolonged period (up to 72 hours, in current practice), the HTC could consider changing the policy to reserve blood only for 24 hours for emergency demands. Also, we can adopt a TS policy as compared to the conventional crossmatch-and-reserve policy at our hospital to further improve blood utilization. These practices will help in reducing the wastage of blood resulting from compromised shelf life due to reservation. In India, still being in the developing stage, where resources are crunch, it is crucial to optimize the resources efficiently, particularly consumables, equipment usage, power supply, and manpower deployment. An additional advantage will be a decreased financial burden on patients due to unnecessary tests including cross-matching and, at times, prolongation of hospital stays. These quality indicators and frequent regular audits will improve blood transfusion safety and act as a tool for developing the best transfusion policies.

As per our current understandings, the limitations of this study include the following: The study was conducted at a single center, thus limiting the generalizations of findings to other healthcare settings. Further, this study lacks in-depth analysis of contributing factors for over-ordering and under-utilization in different surgical departments.

## Conclusions

The retrospective transfusion audit highlights the problems encountered in blood transfusion practices at our center, emphasizing the need for regular audits to ensure optimal utilization of blood resources. The analysis of various quality indicators provides a comprehensive assessment of blood ordering and utilization practices across various specialties and super-specialties.

The study recommends adopting a type-and-screen policy, implementing MSBOS, implementing patient blood management, and revising blood reservation policies to address the identified inefficiencies. Additionally, the involvement of HTCs in developing the best transfusion policies is also emphasized. By implementing these recommendations, healthcare institutions can enhance blood transfusion safety, optimize resource utilization, and ultimately improve patient care while reducing financial burdens and unnecessary wastage.

Overall, the study emphasizes the critical role of continuous quality improvement and vigilant monitoring in ensuring the effectiveness and safety of blood transfusion practices.
